# Consultation on UTUC, Stockholm 2018 aspects of diagnosis of upper tract urothelial carcinoma

**DOI:** 10.1007/s00345-019-02732-8

**Published:** 2019-03-26

**Authors:** Grzegorz Fojecki, Anders Magnusson, Olivier Traxer, Joyce Baard, Palle Jörn Sloth Osther, Georg Jaremko, Christian Seitz, Thomas Knoll, Guido Giusti, Marianne Brehmer

**Affiliations:** 1 Department of Urology, Hospital of Southern Jutland, Sønderborg, Denmark; 2 Department of Radiology, University Hospital, Uppsala, Sweden; 3 Hôpital Tenon, Sorbonne Université, Paris, France; 4 Department of Urology, Amsterdam UMC, University of Amsterdam, Amsterdam, Holland; 5 Urological Research Center, Lillebaelt Hospital, University of Southern Denmark, Vejle, Denmark; 6 Department of Clinical Pathology and Cytology, Karolinska University Hospital Solna, Stockholm, Sweden; 7 Department of Urology, Medical University of Vienna, Vienna, Austria; 8 Department of Urology, Teaching Hospital University Tuebingen, Sindelfingen, Germany; 9 Department of Urology, IRCCS San Raffaele Hospital, Ville Turro Division, Milan, Italy; 10 Division of Urology, Department of Clinical Sciences, Danderyd Hospital, Karolinska Institutet, Stockholm, Sweden

**Keywords:** Upper tract urothelial carcinoma, UTUC, Diagnostics, CT urography, Ureteroscopy, Diagnostic samples

## Abstract

**Purpose:**

To summarize knowledge on upper urinary tract carcinoma (UTUC) regarding diagnostic procedures, risk factors and prognostic markers.

**Methods:**

A scoping review approach was applied to search literature in Pubmed, Web of Science, and Embase. Consensus was reached through discussions at Consultation on UTUC in Stockholm, September 2018.

**Results:**

Tumor stage and grade are the most important prognostic factors. CT urography (CTU) including corticomedullary phase is the preferred imaging modality. A clear tumor on CTU in combination with high-grade UTUC in urine cytology identifies high-risk UTUC, and in some cases indirect staging can be obtained. Bladder urine cytology has limited sensitivity, and in most cases ureterorenoscopy (URS) with in situ samples for cytology and histopathology are mandatory for exact diagnosis. Image-enhancing techniques, Image S1 and narrow-band imaging, may improve tumor detection at URS. Direct confocal laser endomicroscopy may help to define grade during URS. There is strong correlation between stage and grade, accordingly correct grading is crucial. The correlation is more pronounced using the 1999 WHO than the 2004 classification: however, the 1999 system risks greater interobserver variability. Using both systems is advisable. A number of tissue-based molecular markers have been studied. None has proven ready for use in clinical practice.

**Conclusions:**

Correct grading and staging of UTUC are mandatory for adequate treatment decisions. Optimal diagnostic workup should include CTU with corticomedullary phase, URS with in situ cytology and biopsies. Both WHO classification systems (1999 and 2004) should be used to decrease risk of undergrading or overtreatment.

## Introduction

Upper tract urothelial carcinoma (UTUC) has an incidence of less than two cases per 100 000 in the Western world [1]. About 60% of tumors are invasive at the time of diagnosis [2]. Radical nephroureterectomy (RNU) was the standard treatment of UTUC but the European Association of Urology (EAU) guidelines in 2018 recommend that kidney-sparing surgery should be discussed in cases of low-risk UTUC. The EAU guidelines define low-risk UTUC according to these criteria, all of which must be fulfilled: unifocal disease, tumor size < 2 cm, low-grade cytology and histology (biopsy), and no invasive aspects on computed tomography urography (CTU). High-risk indicators are any of the following: hydronephrosis, tumor size > 2 cm, high-grade cytology and histology (biopsy), multifocality, previous radical cystectomy for bladder cancer, and variant histology. The risk factors are based mainly on retrospective reports, and the level of evidence is low. The EAU guidelines use the 2004 WHO classification system for grading tumors [3].

Grade and stage are the strongest prognostic factors [4, 5], whereas tumor size and multifocality may be less significant for the disease-specific survival [6]. Accordingly, correct grading and staging are highly important. The EAU guidelines for diagnostic workup include cystoscopy, CTU, ureterorenoscopy (URS) and biopsy in cases in which additional information can have an impact on treatment decisions [3].

This review aims to consider the challenges in achieving correct diagnosis of UTUC by discussing the different steps in diagnostic procedures, expanding the current recommendations, and showing directions for future research. A scoping review approach was applied to search literature in Pubmed, Web of Science, and Embase.

## Diagnostic workup

### Radiological workup

CTU has higher sensitivity than intravenous urography for diagnosing UTUC [7]. In patients with contraindication for use of contrast medium, magnetic resonance urography can be an option.

Studies have underlined the importance of the manner in which CTU is performed [8, 9]. Helenius et al. [10] showed that enhancement of urothelial carcinomas was highest in the corticomedullary phase (CMP) and, in a subsequent investigation [11] confirmed that, compared to other phases, the CMP offered superior sensitivity and negative predictive value for detection of urothelial cell carcinoma. Metser et al. [12] concluded that the urothelial phase was better than the excretory phase for detecting UTUC. In a prospective study, Grahn et al. [13] observed that multiphase CTU including a CMP offered 85% sensitivity in detecting UTUC. The sensitivity dropped to 58% when a non-optimized CT was performed. When comparing sensitivity and specificity of ureterorenoscopy (URS) and multiphase CT in UTUC, Grahn and co-workers found evidence that multiphase CTU might even provide higher sensitivity than endoscopy, although the specificity was significantly higher for URS. Tumors the researchers described as being difficult to visually diagnose at URS were carcinoma in situ (CIS), which has also been reported by other authors [14].

Current radiological methods cannot diagnose invasiveness unless the tumor is advanced. However, for staging, CT scan is informative regarding lymph node involvement or distant metastasis.

**Fig. 1:**
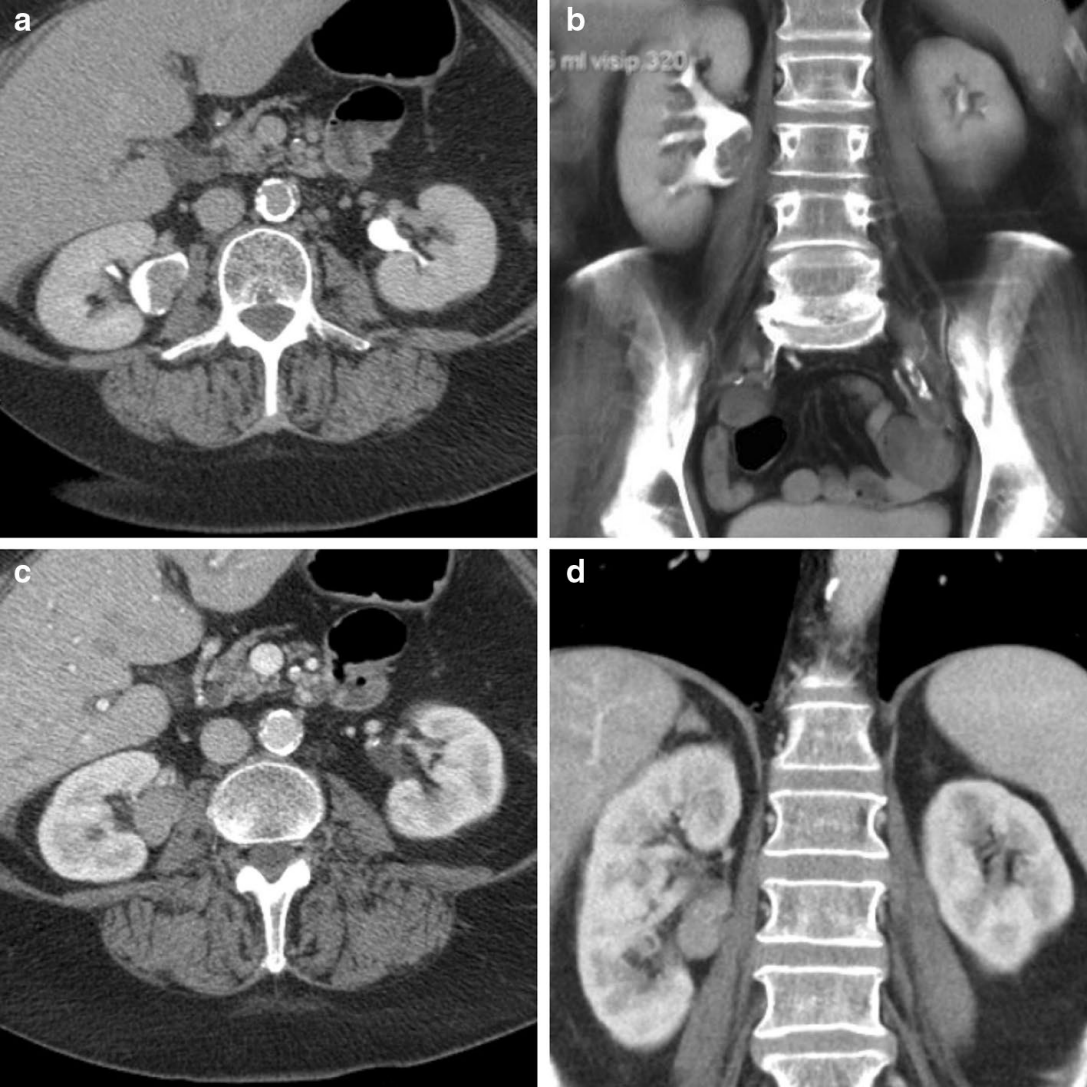
Mutiphase CT urography of a tumor in the right renal pelvis. In the excretion phase (**a**, **b**), a filling defect is clearly visible in both the axial and the coronal plane, although this defect may have been caused by a blood clot. In the corticomedullary phase (**c**, **d**), the lesion is contrast enhanced, proving that there is tumor growth

In conclusion, CTU including CMP is the preferred imaging modality in the diagnostic workup of UTUC (Fig. 1).

### Ureterorenoscopy

The emergence of thinner instruments for flexible URS (fURS) with digital technology has improved the diagnostic value of URS. Using image enhancement techniques during URS can improve detection of urothelial tumors.

The Image1 S™ system (Karl Storz Endoscopy) modifies white-light (WL) images into four different imaging modalities designated as Spectra A, Spectra B, Chroma, and Clara. The Spectra A modality highlights the contrast of capillaries and vessels in the mucosa, Spectra B modality reduces the red spectral reflection highlighting the contrast between tissues and structures, Chroma enhances the sharpness, and Clara enhances local brightness adaptation in the image with clearer visibility of darker regions. The modes can be activated simultaneously in a combined Clara & Chroma mode (C&CH). Kamphuis et al. [15] compared the performance of C&CH with that of WL cystoscopy, investigating 80 bladder tumors, and concluded that C&CH and Spectra B offered the best tumor visualization. Emiliani et al. [16] used standardized templates to assess image quality with regard to color contrast and image definition of Flex XC (Karl Storz, Tüttlingen, Germany). Clara and C&CH scored better than WL (*p* = 0.0139 and *p* < 0.05, respectively), but Spectra A and Spectra B scored worse than WL (*p* = 0.0005 and *p* = 0.0023). Using the same standardized template, Talso et al. [17] compared seven different fURS systems (Lithovue, Olympus V, Olympus V2, Storz Flex XC, Wolf Cobra vision, Olympus P6, and Storz Flex X2) and found that digital scopes offered better image quality than fiber optics. The best overall performance was provided by Flex XC with C&CH (*p* < 0.0001). Clinical studies documenting the value of Image1 S technology are needed.

Narrow-band imaging (NBI) (Olympus Surgical, Orangeburg, NY) is an optical image enhancement technology narrowing the wavelength to 415 and 540 nm, corresponding to blue and green light, respectively. This specific light is absorbed by hemoglobin, thereby increasing the visibility of capillaries at different depths. A meta-analysis (including > 1000 patients) demonstrated that, compared with WL, NBI achieved up to 24% higher detection of bladder tumors [18] and offered greater sensitivity both per person and per lesion [19].

Two prospective studies have evaluated NBI for detection of UTUC. Traxer et al. [20] performed URS on 27 patients with UTUC, applying WL and NBI; Compared with WL, NBI detected five additional tumors and also revealed extended tumor growth in three cases. Hao et al. [21] obtained nearly identical results in 54 fURS examinations performed on 16 patients. The studies show that NBI may enhance visualization of tumors and potentially improve both diagnostics and treatment of UTUC.

### Biopsy for histopathology

Tumor grade and stage are key factors in risk stratification of UTUC. Therefore, biopsy for histopathology is an important part of the diagnostic procedure. There is no consensus regarding which biopsy method is preferable to achieve representative samples [22]. Harvesting good-quality tissue samples was accomplished in only 75% of cases in a study by Tavora et al [23]. In a consecutive cohort of patients who underwent RNU at three centers in the United States in 2000–2016, initial biopsy, using cold cup forceps or basket, underestimated tumor grade in 18% of cases [24]. There was significant discordance between URS biopsy and final pathology for UTUC: positive predictive values (PPVs) for invasiveness of high grade on biopsy was 60%; negative predictive values (NPVs) for invasiveness of low grade on biopsy was 80%. Smith et al. [25] documented even greater discrepancy between biopsy and final pathology of RNU specimens: 43% (24/56) of the tumors were upgraded after RNU. Limitations of the biopsy technique and tumor heterogeneity are possible explanations of initial underestimation of grade and stage [26].

Margolin et al. [24] noted that the likelihood of missing invasion on URS biopsy was significantly increased when the diameter of biopsy fragments was ≤ 1 mm (OR 4.3). To overcome this limitation, larger biopsy forceps (10 Fr cup), BIGopsy (COOK Medical, USA), have been developed. Three studies [27–29] compared BIGopsy forceps with standard biopsy techniques and nitinol baskets, and concluded that BIGopsy retrieved biopsies more appropriate for histopathological evaluation. However, they also identified disadvantages of BIGopsy, including decreased visibility and the necessity of using ureteral access sheaths (UASs) for backloading into the endoscope. Basket biopsy is excellent for exophytic tumors, whereas cup forceps are most suitable for flat lesions [28, 29].

Although biopsy quality may be improved by a larger biopsy device or a multi-biopsy approach [30], it has been suggested that addition of cytology should be mandatory to improve grading [24]. Messer et al. [31] showed that positive urinary cytology had sensitivity of 56% and a PPV of 54% for high-grade UTUC, and corresponding values of 62% and 44% for muscle-invasive UTUC; using selective upper tract cytology increased the PPV for detecting high-grade lesions to 85%. In a meta-analysis of the diagnostic utility of selective upper tract urinary cytology [32], pooled sensitivity of selective cytology with respect to final pathology was 53.1%, and corresponding values for low- and high-grade tumors were 45.6% and 69.9%, respectively. Thus, selective cytology alone is not perfect for grading and staging UTUC. In a prospective study of 51 patients, Keeley et al. [33] used both biopsy and cytology. Fresh samples for cytology were taken in 48 subjects, whereas biopsy material was sufficient in 42 of those cases. This approach increased the concordance rate with final pathology to 90% for low–moderate-grade lesions and to 91.6% for high-grade lesions. Importantly, there was significant correlation between grade and stage in this study. Williams et al. [34] obtained similar results when using a nearly identical protocol in a retrospective analysis.

Similarly, in a study of a prospective series of UTUC patients (*n* = 43), Malm et al. [35] used a strict protocol comprising bladder barbotage before manipulation, non-touch URS, renal pelvis barbotage, and collection of fluid in the bladder after URS to detect ureteral tumors. With this approach, Malm and colleagues showed that cytology was as effective as final pathology to identify malignancy and to assess grade. Hence, it seems that strict sampling protocols including cytopathological evaluation of fresh samples may increase sensitivity for both grading and staging.

### Confocal laser endomicroscopy

Confocal laser endomicroscopy (CLE) enables real-time visualization of tissue microarchitecture and cellular morphology. A fluorescent contrast agent that stains extracellular matrix of the tissue is applied in the upper urinary tract and a mobile laser (488 nm) scanning unit with a fiber optic probe is used to scan the tissue. In the urinary tract, CLE has mainly been used to distinguish between low- and high-grade lesions in the bladder [36]. Three reports have discussed the feasibility of applying CLE in the upper urinary tract [37–39] for grading of UTUC. All three investigations were based on small case series but nonetheless show high concordance with low-grade UTUC and CIS on final pathology. Most of the studied CLE specimens were compared with biopsy samples and not with RNU specimens, limiting the validity of the observations. The technique does seem promising but must be further evaluated.

### Grading and staging

Grade and stage are the strongest prognostic factors in UTUC [3–5]. Direct staging of biopsies is difficult, since samples seldom contain tumor base with lamina propria [22]. However, there is a strong correlation between stage and grade [3, 40]. When using the 2004 WHO classification, low-grade tumors are more likely to be regarded as superficial, whereas it is likely that high-grade tumors will be identified as invasive. The stage–grade correlation seems to be greater for UTUC than for bladder cancer. With the 1973/1999 WHO system, grading tumors as G1–G3, the correlation is very strong for G1 (superficial) and G3 (invasive) tumors, but is more unpredictable for G2 lesions [4, 22, 34]. Brown et al. [41] conducted a retrospective study of 184 UTUC patients undergoing RNU and found that 100%, 71.7%, and 33.8% of G1, G2, and G3 tumors, respectively, were superficial. Maruschke et al. [7] reported similar results, although a larger proportion (88%) of the G3 tumors was invasive. Holmäng and Johansson [42] recut and re-evaluated 555 UTUC specimens from patients with no previous or concomitant bladder cancer. They concluded that the 1973/1999 WHO classification is superior to the 2004 WHO system for clinical use since the correlation between stage and grade was strong when using the 1973/1999 WHO system, but was poor with the 2004 WHO classification. Using the 1973/1999 WHO system, the correlation with stage was strong for G1 and G3 tumors, but was less pronounced for G2 tumors, which may be important when deciding treatment modality [5]. In addition, the disease-specific survival differed between G1 and G2 tumors with the 1973/1999 WHO, an observation that was difficult to assess with the 2004 WHO system.

### Histopathological considerations

**Fig. 2:**
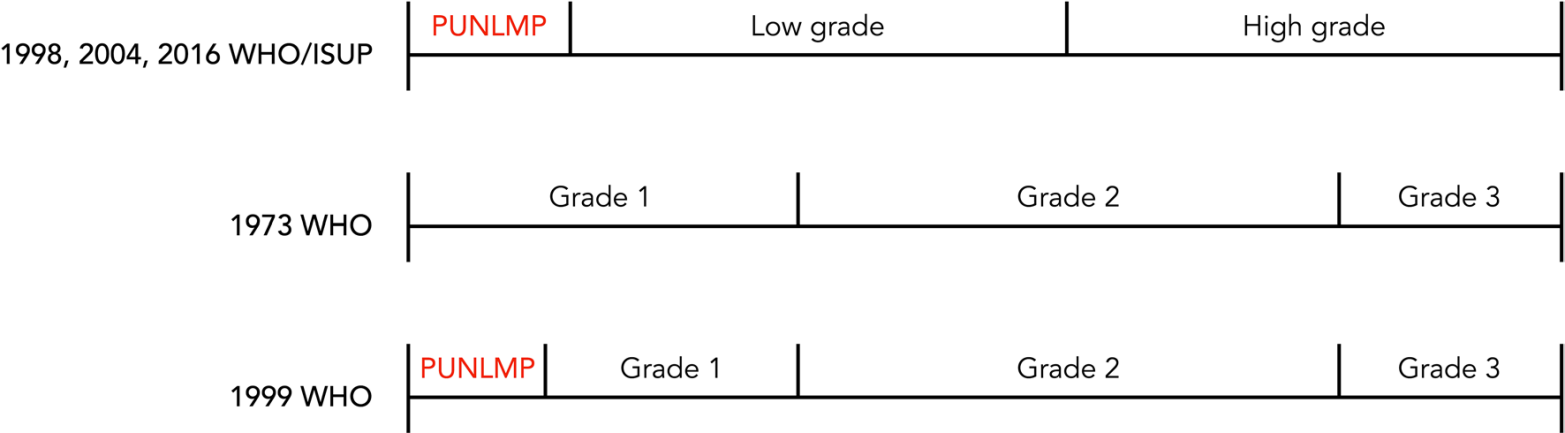
WHO classification systems for grading of urothelial tumors. *PUNLMP* papillary urothelial neoplasm with low malignant potential

The 1973 and 1999 WHO systems for grading of urothelial tumors have been the most extensively used and widely accepted approaches [43]. However, more detailed grading also entails the risk of greater interobserver variability. G2 is most difficult to assess, which has led to the ambiguous use of diagnostic categories such as “grade 1–2” and “grade 2–3”. There has also been confusion regarding G2 (pTa) tumors in that such cases can include high-risk disease. These issues led to introduction of the 1998 WHO/ISUP classification, later revised and presented as the 2004 and the 2016 WHO systems. The three-tiered 1999 WHO classification was divided into a low- and a high-grade category with the intention of ensuring that most patients with high-risk disease would be sorted into the high-grade category (Fig. 2). The morphological criteria were clearly detailed in an earlier study reported by Malmström et al. [44].

The rationale for using a classification system including only a low and a high grade is supported by the existence of two distinct molecular pathways of neoplastic transformation in urothelial tumors in the bladder [45]. Low-grade tumors have a large proportion of FGFR3 alterations (80%). They have a high recurrence rate but non-aggressive behavior. High-grade tumors have an elevated frequency of TP53. They are associated with a high risk of progression to muscle-invasive disease. The 2004 classification offers the advantage of greater interobserver reproducibility [46]. The EAU recommends that the new system is used together with the WHO 1999 grading classification, because it allows comparison of long-term outcomes at different clinical centers.

### Cytology

The Paris system for reporting urinary cytology has gained broad acceptance [47]. It includes the following diagnostic categories:

1. Non-diagnostic/unsatisfactory
2. Negative for high-grade urothelial carcinoma
3. Atypical urothelial cells
4. Suspicious for high-grade urothelial carcinoma
5. High-grade urothelial carcinoma
6. Low-grade urothelial neoplasm
7. Other: primary and secondary malignancies and miscellaneous lesions

The main objective is to identify high-grade UTUC including all G3 and some G2 tumors. “Atypical urothelial cells” is used only when high-grade urothelial carcinoma is suspected, i.e., when there is an indication of aggressive cancer.

### Molecular markers for UTUC

The difficulties in correct grading of UTUC based on the cytology and biopsies indicate the need for more reliable tumor markers.

In a retrospective study of 83 tumors, Bagrodia et al. [48] observed that only two alterations were uniformly related to high-grade and advanced disease: TP53/MDM2 alterations, associated with poor prognosis; and FGFR3 mutations, associated with more favorable outcome. In another study, Bagrodia et al. [49] also found high concordance in genomic alterations between tumor biopsies and subsequent RNU specimens.

Although many different tissue markers have been investigated, none of them have shown the potential to change clinical practice. Most studies have examined gene malformation in RNU specimens, often within selected patient groups. Published investigations of tissue markers are summarized in Table 1.

**Table 1:** Studies of molecular markers for detection of UTUC

Tissue marker	Year	Study type	No. of patients	Conclusion
Gene malformations [47]	2017	Retrospective	83	TP53/MDM2 pathway alterations and FGFR3 mutations associated with high-grade; FGFR3 associated with more favorable outcome
Gene malformations [48]	2018	Retrospective	39	TERT and FGFR3 mutations observed in 64% of high-grade tumors (ureteroscopic biopsies comparable to RNU specimens)
Ki-67 [49]	2015	Prospective, multicentre	475	Independent predictor of RFS and CSS
HER-2 [50]	2014	Retrospective	171	HER-2 gene overexpression was found in 18.1% of cases and in multivariate analysis was correlated with early tumor recurrence in the bladder
HER-2 [51]	2017	Retrospective, multicentre	732	Multivariate analysis confirmed that HER-2 overexpression was associated with RFS and CSS,
EGFR, Ki-67 [52]	2016	Retrospective	320	An independent risk factor for bladder cancer recurrence after RNU

*RFS* recurrence-free survival, *CSS* cancer-specific survival, *HER*-*2* human epidermal growth factor receptor type 2, *EGFR* epidermal growth factor receptor, *RNU* radical nephroureterectomy

### Potential risks of URS in UTUC

The risks associated with URS in treatment of UTUC can be subdivided into two groups: [1] general risks, including high intrarenal pressure (IRP) and ureteral damage caused by the use of a UAS; and [2] specific risks, including issues related to delaying final therapy and tumor seeding. High IRP resulting in tubular and pyelovenous backflow during URS is the main cause of septic and haemorrhagic complications, as well as access-related complications, because IRP induces peristalsis that can compromise access [50]. High IRP can result in forniceal rupture and potentially also perirenal tumor seeding [50]. Such seeding has been reported to occur along a percutaneous nephrostomy tract in UTUC patients [51], although no studies in the literature have concerned perirenal or intratubular seeding following URS. Nevertheless, it is crucial to maintain low IRP during URS in UTUC to reduce both general and specific risks related to the disease. Prospective data on UAS usage in UTUC are lacking. Gorin et al. [52] have described findings suggesting that UAS can facilitate acquisition of high-quality specimens. This information was compiled in an assessment of a retrospective cohort representing 85 procedures with UAS application performed on 64 patients. Sufficient material was obtained in 90.4% of the cases, and concordance with final pathology was found in 88.6%, and the authors concluded that UAS application was safe in UTUC. However, Lildal et al. [53] showed that ureteral trauma following UAS application is often underestimated. It is even possible that injuries to the muscular coat of the ureter can be missed. Such lesions can serve as nidi for through-the-mucosa tumor cell seeding. Thus, it is still a matter of debate whether use of a UAS during URS in UTUC should be recommended. Data must be compiled regarding the safety of UAS usage in UTUC management.

The potential risk of intravesical recurrence after diagnostic URS is well known, although this aspect has not been found to have an impact on overall, cancer-specific, recurrence-free, or metastasis-free survival [54, 55]. Furthermore, Maruschke et al. [7] and Chitale et al. [56]. studied RNUs that were performed based on radiographic findings and without prior URS. Their results showed that in 5.6% (6/113) and 10.2% (4/39) of cases, respectively, no tumor was found on final pathology, highlighting the considerable risk of overtreatment if URS is not performed in patients with suspected UTUC.

## Conclusions

Diagnostic workup of patients with suspected UTUC comprises the following: CTU, preferably with a CMP, for detection of tumors as contrast-enhancing masses rather than as filling defects; cystoscopy and urinary cytology; fURS with biopsy and renal pelvis barbotage cytology.

URS image enhancement systems, Image1 S and NBI, may improve both diagnosis and outcome of local treatment. General risks of URS must be taken into consideration. It appears that URS does not affect the final oncological outcome in UTUC patients, although this procedure does seem to be associated with an increased risk of intravesical recurrence.

There is a strong correlation between tumor grade and stage, and accordingly grading is crucial. The use of strict sampling protocols including cytopathological evaluation of fresh specimens increases the sensitivity of both grading and staging. Both of the WHO classification systems (1999 and 2004) should be used for grading to decrease the risk of undergrading or overtreatment.

Molecular markers for predicting aggressive UTUC are warranted. Further studies in this field are needed.
